# Analysis of motivations that lead women to participate (or not) in a newborn cohort study

**DOI:** 10.1186/1471-2431-13-53

**Published:** 2013-04-11

**Authors:** Liza Vecchi Brumatti, Marcella Montico, Stefano Russian, Veronica Tognin, Maura Bin, Fabio Barbone, Patrizia Volpi, Luca Ronfani

**Affiliations:** 1 Institute for Maternal and Child Health, IRCCS “Burlo Garofolo”, via dell’Istria, 65/1, 34137, Trieste, Italy; 2 Department of Medical and Biological Sciences, Unit of Hygiene and Epidemiology, University of Udine, Udine, Italy

**Keywords:** Newborn cohort study, Motivation, Participation, Refusal, Withdrawal

## Abstract

**Background:**

Little is known about reasons that influence parents’ decision to participate in studies enrolling healthy children. The aim of this observational study was to verify the reasons that lead pregnant women to give their consent or to refuse participation to a newborn cohort study with a long follow up time.

**Methods:**

To prospectively investigate the reasons that lead women to participate, to refuse the participation or to withdraw from a newborn cohort study, three different questionnaires were administered to pregnant women contacted or enrolled in the Phime cohort study, carried out in an Italian Hospital from 2007 to 2010.

**Results:**

Phime study participation was refused by 304 women and 145 withdrew their consent during the follow up. All these women filled in the related questionnaires. Within 632 mothers in follow up at 18 months, 430 filled in the questionnaire on motivation to participate: 97% stated that the main reason was to contribute to research; 96% and 90% stated that they wanted to benefit future babies’ and mothers’ health. Ninety-six percent of women would appreciate to know the results of analysis carried out on biological samples collected and of the overall study results. One third of the mothers (37%) wanted to be involved in the definition of future similar studies, bringing their experience and their views. Within the 304 women who refused participation, 56% stated that the study was too demanding, 26% was not interested in participating and 18% was concerned about the need to collect biological samples and to be submitted to neurocognitive tests. Fifty-two percent of 145 women who withdrew after enrollment stated that the study was too demanding (52%), and 6% was concerned about the biological samples collection.

**Conclusions:**

The altruistic reason appears to be the main reported by women to decide to participate in a newborn cohort study. The fact that the study was too demanding and the need to collect biological samples are important reasons that lead women to refuse participation or to withdraw from the study. An adequate communication on these aspects should minimize difficulties in enrolment and losses to follow up.

## Background

Medical progress is closely linked to research. Clinical studies such as randomized controlled trials or cohort studies are crucial to increase knowledge about effective interventions, risk and protective factors, prognosis of diseases. The success of clinical studies depends on the adequate enrollment of healthy or sick subjects, on their compliance to the study protocol and on the completeness of the follow up. The expectation of people to receive adequate medical care often contrasts with the lack of awareness of the importance to participate in clinical research. This is true particularly when healthy people are asked to participate to studies or when studies involve relative or family members [1]. In the case of children participation to studies, parents are almost always called to give consent. In this context, it is crucial to understand what reasons influence parents’ decision. Available evidence on this topic are scanty. Studies carried out in sick children show that the most important reasons influencing parents’ decision are learning more about their child’s illness and altruistic reasons (i.e. to contribute to medical knowledge or to help others) [2-4]. Other factors highlighted in the scientific literature are the opportunity to have benefits (i.e. financial benefits, free or new medications, gifts), the duration of the study and where it is carried out, parents’ attitude towards clinical trials and research, the risk/benefit assessment and the socioeconomic status [2,5-7].

The case in which researchers enroll healthy children or parents of children before birth is more complex. This happens, for example, in newborn cohort studies, in which usually women were approached during pregnancy and the mother-child dyads are followed up for years. This study design is particularly hard for parents and their children, due to the long follow up, the need to fill in very detailed questionnaires, the periodic child evaluations in hospital or in outpatient setting, the collection of biological samples. On this topic only studies investigating the theoretical willingness of mothers to participate to clinical research are available [8,9]. Mothers were asked to imagine their children involved in different studies’ scenarios with different level of risk, discomfort and benefit. The results showed that the willingness to consent is greater if the child is healthy and the research is not intended to solve a child’s specific problem. The degree of consent was inversely proportional to the risk. The altruistic reason was the main indicated by mothers to consent children participation to high risk studies. To the best of our knowledge, no field study assessing parents motivation to accept or to refuse participation in newborns cohort studies was carried out.

The aim of this observational study was to verify the reasons that lead healthy pregnant women to give their consent or to refuse participation to a newborn cohort study with a long follow up time. The results would contribute to improve the design and conduction of observational clinical studies and the communication to parents.

## Methods

This survey was carried out at the Institute for Maternal and Child Health IRCCS Burlo Garofolo of Trieste (Italy), a third level hospital and one of the three pediatric research institute recognized by the Italian Ministry of Health.

The mothers of healthy term newborns contacted or enrolled in the Phime cohort study (Public health impact of long-term, low-level mixed element exposure in susceptible population strata, 6th Framework Program) were asked to participate in this survey. The protocol of the Phime study was approved February 5, 2007 by the Bioethics Committee of the Institute for Maternal and child Health IRCCS Burlo Garofolo. An amendment, including a prolonged follow up (up to 7 years of child’s life) and the possibility to re-contact parents in order to carry out studies related with the Phime (such as the present), was approved April 12, 2009. Subsequently, women were contacted during the follow-up visits to give their consent to fill-in the questionnaires about motivations. The informed consents for both the Phime and the motivations studies were written and obtained face to face by two trained researchers.

The Phime study assessed the association between low-level prenatal mercury exposure through maternal fish consumption and child neurodevelopment [10]. The first results of the study are published elsewhere [11]. The participation to the Phime study was very demanding for families. All the women living in the area of Trieste, ≥18 years of age and Italian speaking were contacted during pregnancy. After enrollment, at 20^th-^22^nd^ week of pregnancy (morphological ultrasound screening), a blood sample was collected, a hair sample was cut from the occipital area of the scalp and a brief questionnaire about food habits, socio-demographic information and health status was filled in; at 30^th^-32^nd^ week of pregnancy (biometric ultrasound screening) the Raven’s standard progressive matrices test of intellectual efficiency and general intelligence was administrated to mothers and a urine sample was collected; at delivery a cord blood sample, a piece of cord tissue and, when possible, a sample of the first urine of the newborn were collected; within the first month of life, a more extensive questionnaire, including detailed food frequency questions and the evaluation of environmental exposures, was filled in by mothers; a 24 hour breast milk collection was carried out one month after birth; in the second year of life, the child postnatal dietary exposure, including types and amounts of food consumed, was recorded by parents through a one week diary; at 18 months after birth, a home visit was carried out to evaluate the familiar environment using the HOME (Home Observation for Measurement of the Environment) questionnaire; finally, to evaluate the child neurocognitive abilities at 18 and at 40 months of life the Bayley Scales of Infant and Toddler Development, Third edition (BSID III) was administered during an *ad hoc* hospital visit.

The Phime study started the enrolment of women during pregnancy in April 2007; the 18 months follow up started in January 2009.

To evaluate the reasons that led women contacted for the enrollment in the Phime cohort study to give or not their consent to participate, two different questionnaires were administered:

1) Women who give their consent: after the evaluation of child neurocognitive abilities at 18 months of life (BSID III), mothers were asked to fill in an anonymous questionnaire about motivation to participate, consisting of multiple choice questions investigating four areas: 1) the reasons influencing the participation to the Phime study; 2) the possible reasons affecting the choice to participate to further studies or to refuse the participation; 3) the appropriateness of the enrollment place; 4) the involvement during pregnancy in other studies other than Phime. Socio demographic information (women age, years of education, marital status, working situation) were also collected. This questionnaire was not part of the Phime study and was developed ad hoc. After receiving the approval of the Bioethical committee in April 2009, it was proposed to all mothers enrolled in the Phime study and in follow up at 18 months. A simplified version of the questionnaire is available as Additional file 1.

2) Women who refused to participate or withdraw their consent: these questionnaires were part of the Phime study and they were administered to women at the time of refusal or withdrawal. They include multiple choice questions about the reasons which influenced the refusal or the withdrawal and about socio demographic characteristics.

The responses to the questionnaires were entered into a database using EPIDATA (http://www.epidata.dk).

A descriptive analysis was carried out; categorical data are presented as absolute frequencies and percentages; continuous data as medians and interquartile ranges (IQR). To compare the main socio-demographic variables available in questionnaires (women age at delivery and level of education) among groups, the Kruskal-Wallis test and the Fisher exact test were used respectively. To evaluate the potential influence of socio-demographic characteristics (women age, education, marital status, working situation) on the willingness to participate to further research in women who filled in the questionnaire on motivation to participate, a multiple logistic regression analysis was carried out.

## Results

One thousand five hundred ninety five women were contacted during pregnancy; three hundred ninety-one resulted not eligible (i.e., due to language barriers or hair length insufficient for sample). Among the remaining 1204 women, 900 (75%) were enrolled and 304 refused to participate. Seven hundred sixty-seven of the enrolled women (85%) remained in the study at delivery and 632 children underwent BSID III testing at 18 months (70% of the initial cohort). Overall, 268 of 900 women (30%) left the study after enrollment: 50 became not eligible (i.e., miscarriage, preterm birth, severe mother or child intercurrent illness, etc.), 145 withdrew their consent to continue the follow up and 73 were lost to follow up. Four hundred and thirty mothers of the 632 children followed up at 18 months (68%) accepted to fill in the questionnaire on motivation to participate.

The main characteristics of women which filled in the questionnaires about motivation to participate (n = 430), motivation to refuse (n = 304) and motivation to withdraw (n = 145) are described in Table 1. Women who withdrew participation presented a statistically significant lower level of education.

**Table 1:** Characteristics of women filling in the three questionnaires

	**Motivation to participate questionnaire (n = 430)**	**Motivation to refuse questionnaire (n = 304)**	**Motivation to withdraw questionnaire (n = 145)**	**p value**
**Median age, (IQR)**	33.8 (31.0-36.7)	34.6 (31.0-36.4)	33.8 (30.0-36.3)	0.6
**Level of education, n (%)**				
- Elementary/Middle school	62 (14.4)	46 (15.1)	37 (25.5)	0.008*
- High school /University degree	368 (85.6)	258 (84.9)	108 (74.5)	

* significant differences between first and third columns and between second and third columns (Fisher exact two tailed test, respectively p = 0.003 and p = 0.009).

**Questionnaire on motivation for participation**: Table 2 describes the results concerning the reasons that led mothers to participate to the Phime study. The most frequent answer given by mothers was to contribute to research and science (97%). Mothers interviewed had the correct perception that study participation would not have been beneficial for them or for their babies but only for future mothers and babies (90% and 96% respectively).

**Table 2:** Reasons that lead women to participate to the cohort study (n = 430)

To contribute to the research and science	97%
To consent that other children can take advantage from the results of this study in the future	96%
To consent that other mothers can take advantage from the results of this study in the future	90%
Study proposed by an institution in which I trust	78%

Almost all of the mothers (99%) stated that they felt free to participate. Thirty-six percents stated that the study participation was too time consuming, especially with respect to filling in the alimentary diary.

Eighty-two percent of mothers answered that they would participate to further research projects to contribute to research and science (96%) (Table 3). The remaining 18% stated that they would not participate mainly for the time and the involvement required (92%) and for the need to collect biological sample of the child (44%). At multivariate analyses, socio demographic variables (age, marital status, years of education, working situation) and being involved in other studies did not influence the decision to participate to further researches.

**Table 3:** Reasons that lead women to participate to further cohort studies or projects (n = 430)

To contribute to research and science	96%
To consent that other children can take advantage from the results of these studies in the future	95%
To consent that other mothers can take advantage from the results of these studies in the future	91%
Study is proposed by a institution in which I trust	74%

Eight percent of the interviewed women stated that the time allowed to make a decision was too short and 6% that the place where the enrollment took place was inadequate (not comfortable, too small, without privacy).

Ninety-six percent of women would appreciate to know the results of analysis carried out on biological samples collected and of the overall study results. One third of the mothers (37%) wanted to be involved in the definition of future similar studies, bringing their experience and their views.

**Questionnaire on motivation for refusal**: at enrollment, 304 pregnant women refused to participate to the Phime project: 56% stated that the study was too demanding and that they had no time to participate, 26% was not interested to participate and 18% was concerned about the need to collect biological samples and to be submitted to neurocognitive tests.

**Questionnaire on motivation for withdrawal**: this questionnaire was filled in by 145 women who withdrew after the enrollment (84 during pregnancy and 61 after birth). The following reasons were stated to motivate the withdrawal: study too demanding (52%), no more interest in participating (14%), intercurrent health problems for the women or for the child (12%), opposition to the biological samples collection (6%), other reasons (15%) such as back to work, personal reasons, etc.

## Discussion

Newborn cohorts are the most appropriate study design to determine causation between risk or protective factors and child health. Unfortunately, it is very expensive to conduct, since it usually involves researchers and participants for many years. Moreover, the long follow up puts the cohort at risk of significant losses that lead to a reduction of internal and external study validity. In literature, little is known about the real causes that lead to these losses. The results of this observational study confirm the difficulties in enrolment and the risk of loss to follow up related to newborn cohort studies: only 900 of 1204 women eligible (75%) decide to participate and, of these, only 70% were in follow up 18 months after child birth. A relevant number of women was lost to follow up or withdrew their consent (24%). Women who withdrew the consent presented a lower level of education while no differences were found between women who refused and women still in follow up at 18 months. This finding may be due to a higher propensity in accepting to participate in the study without enough conviction in women with lower educational level. There were no differences in the age of the women enrolled in the three groups. The main reasons reported by women to justify the non-participation or the withdrawal from the study are common to these two groups: the newborn cohort study is considered too demanding, requiring too much time and a high level of involvement; women do not have any interest in participating or this interest declines during the follow up period; there is concern about possible risks associated with the collection, the long-term storage, the future use of biological samples and with the need to expose a child seen as “healthy” to long and stressful neurodevelopment tests. In addition to this data, it is important to highlight that almost 20% of women still in follow up at 18 months stated that they were no longer available to participate in further research projects. In this population too, after child birth, concerns about collection and long-term storage of biological samples became an important reason to refuse future participation in cohort studies.

The altruistic reason appears to be the main reported by women to decide to participate in the Phime study. Almost all the enrolled women stated they would contribute to research and science and to the health of other mothers and children. This data confirms the results shown by studies involving sick children [2-7].

The identification of the main causes that lead women to participate to newborn cohort studies, to refuse the enrolment or to withdraw the consent during follow up allow researchers to develop strategies to minimize losses. To achieve this goal, it is not possible to act by modifying the study design or the follow up duration, which are predetermined and linked to study objectives. However, through the analysis of the answers provided by women in the present study, some possible areas of work can be identified:

Given the importance of the altruistic reasons to ensure women participation in newborn cohort studies, the possible benefits for future mothers and children would be emphasized in the informed consent and during the communication between researchers and women at enrolment.

Correct and detailed information about the collection, the long-term storage, the future use of biological samples is also important. Literature on this topic confirms the findings of the present paper. Maternal attitudes toward DNA collection in epidemiological studies was assessed through focus groups [12,13]. Distrust of the government, fathers’ skepticism, and insufficient information about the use, storage, and disposal of specimens were reported as primary barriers to participation in DNA collection. Addressing these concerns in study materials might increase participation rates. Altruism was reported as the primary reason for participation. Noninvasive methods for DNA collection or the use of residual samples were preferred by most mothers. Parents concerns about storage of residual newborn screening samples for future use were also addressed in literature [14-16]. If the samples were stored and used for biomedical research, stakeholders involved in focus groups stated that an adequate informed consent would be needed with some type of institutional review board approval for protection [17]. The importance of considering parental permission in storage of residual newborn screening samples is confirmed by a survey of a nationally representative sample of USA parents: over three-quarters of parents interviewed would be willing to permit the use of their children’s samples for research purposes if their permission was obtained prior to such use. However, if permission was not obtained, more than half of the parents would be ‘very unwilling’ to permit use of their child’s sample [18].

These evidences from literature and the answers given by mothers in the present study, address the importance of an adequate and detailed information to parents. Communication would occur during the prenatal period [19] and would stress that 1) samples will be collected so as to cause the least possible disturbance to the child (for example during procedures already scheduled for clinical reasons); 2) their storage will be adequate, following pre-determined procedures and the national legislation on bio-banking; details on how long the samples will be stored and on what will happen to the samples after the end of the study would be provided; 3) the anonymity of the samples will be provided through the use of appropriate coding; 4) the samples will be used exclusively for the purpose of the study for which they were collected. All this information would be included in the informed consent form.

A percentage of women indicated as critical their insufficient involvement both in the study design phase and during the follow up period. Women stated that the knowledge of results of tests carried out and of the overall study results should be appreciated and useful to motivate participation. Literature on participatory research, that is the co-construction of research between researchers and people affected by the issues under study, provides evidences that co-governance can be beneficial to research contexts, processes and outcomes, including increased recruitment and response rate [20,21]. A more participatory approach in designing cohort studies would allow researchers to work with women and other stakeholders to select and justify the strongest possible research methods, while balancing research rigor with their responsiveness to the community; emerging problems during the enrolment phase and the follow up period could be discussed and solved with the stakeholders. In our setting, a more participatory approach in the Phime study would have been possible, for example involving groups of women who access the antenatal classes carried out by primary care midwives. In the Phime study, researchers tried to make the enrolled women more involved in the study activities through periodical letters with news and results. This approach was not considered satisfactory by some of the women enrolled. The creation of a website and the periodical sending of SMS or e-mails could strengthen the communication with women after enrolment. A more active participation could be obtained by using social networks, such as Facebook or MySpace. This approach would allow women to have a direct and real-time contact with researchers and, even more important, with other women enrolled. In literature, evidences of the use of social networks for clinical purpose (i.e. patient-physician relationship, health promotion programs) and for research (i.e. public health research) were found [22-25]. A recent paper addressing the role of social networks in recruiting adolescent girls into a follow up study, showed that loss to follow up was minimized by contacting potential participants through Facebook. In this study, researchers tried to re-contact girls enrolled in a previous trial on physical activity to re-recruit them in a follow up study. Through Facebook, the researchers were able to locate 9% and ultimately re-recruit 6% of the participants of the previous study who they would otherwise not have been able to reach using the traditional recruitment methods [26]. The use of social networks to fidelize women enrolled in newborn cohort studies has never been explored.

## Conclusions

This study, carried out in women contacted or enrolled in a birth cohort study, made it possible to draw a complete picture of motivation that lead women to participate, to refuse the enrolment or to withdraw the consent during follow up. This information allowed to identify some possible areas to work on to try to minimize difficulties in enrolment and to reduce the risk of loss to follow up. A clear and adequate communication (without hiding anything) with women during pregnancy, addressing the possible benefits for future mothers and children that may result from their participation in the study and the management of some crucial steps of the study (such as the collection, storage and use of biological samples), is decisive both to convince women to participate and to remain in follow up. A more participatory approach in designing the studies and a more active participation of women during the follow up period should contribute to reduce the losses. In this, a role of Internet and in particular of social networks, such as Facebook, is conceivable but it is still to be verified.

## Competing interests

The authors declare that they have no competing interests.

## Authors’ contributions

LVB conceived of the study, performed the field research, contributed to interpretation of data and drafted the manuscript. MM performed the statistical analysis. SR contributed to draft the manuscript. VT contributed to acquisition of data. MB contributed to acquisition of data. FB contributed to conception and design. PV contributed to draft and to review the manuscript. LR conceived of the study, contributed to conception and design, participated in coordination and drafted the manuscript. All authors read and approved the final manuscript.

## Pre-publication history

The pre-publication history for this paper can be accessed here:

http://www.biomedcentral.com/1471-2431/13/53/prepub

## Supplementary Material

Additional file 1 A simplified version of the questionnaire on motivation for participation. Click here for file
