# Supplementary material for: Analysis of motivations that lead women to participate (or not) in a newborn cohort study
Source: BMC Pediatr. 2013 Apr 11;13:53. doi: 10.1186/1471-2431-13-53 (PMC3636025; doi:10.1186/1471-2431-13-53)
Supplement: Additional file 1 — A simplified version of the questionnaire on motivation for participation. [file 1471-2431-13-53-S1.pdf]

**Additional file 1. A simplified version of the questionnaire on motivation for participation.**

| |
|---|
| Why have you participated to the Phime research project?<br>Because:<br>- it has been proposed by an institution in which I trust<br>- to get medical benefit for myself<br>- to get medical benefit for my baby<br>- to get more attention by the personnel<br>- to get my baby more attention by the personnel<br>- to consent that other mothers can take advantage from the results of this study in the future<br>- to consent that other children can take advantage from the results of this study in the future<br>- to contribute to research and science<br>- for other reasons |
| Have you felt forced to participate? |
| Which is the main advantage to participate? |
| Which is the main disadvantage to participate?<br>- it has been proposed by an institution in which I trust<br>- to get medical benefit for myself<br>- to get medical benefit for my baby<br>- to get more attention by the personnel<br>- to get my baby more attention by the personnel<br>- to consent that other mothers can take advantage from the results of this study in the future<br>- to consent that other children can take advantage from the results of this study in the future<br>- to contribute to research and science<br>- for other reasons |
| After this experience would you participate in other studies? |
| if not, why?<br>- no advantage for myself<br>- no advantage for my baby<br>- time and involvement requested<br>- biological samples of my baby<br>- neurological/psychological tests on my baby<br>- because the partner objects<br>- depends on the studies |
| Do you think you had enough time to take the decision? |
| Was the place where the participation was proposed adequate?<br>if not, why?<br>- not enough privacy<br>- place not cozy<br>- place too small |
| Would you want to know the results of the tests?<br>Would you want to know your baby's tests results?<br>Have you known the results of the tests?<br>Have you known your baby's tests results?<br>Would you like to know further details about the results of the study?<br>Would you like to be involved in a new research study to offer your experience, opinion? |

Vecchi Brumatti L, et al. Why should my baby and I take part in this study?

| Socio-demographic data |
| --- |
| - age<br>- marital status<br>- years of education<br>- employment (at the moment of the enrollment)<br>- job category |
